# Supplementary material for: Effects of scapular-focused movement-based exercises on sports performance of athletes with scapular dyskinesis: A systematic review
Source: PLoS One. 2026 Apr 10;21(4):e0344540. doi: 10.1371/journal.pone.0344540 (PMC13068284; doi:10.1371/journal.pone.0344540)
Supplement: S2 File — (DOCX) [file pone.0344540.s002.docx]

**Supplementary File 2 (Reasons for exclusion of studies)**

| **Study** | **Reason for exclusion** |
| --- | --- |
| Gulpinar, D., Ozer, S.T. and Yesilyaprak, S.S., 2019. Effects of rigid and kinesio taping on shoulder rotation motions, posterior shoulder tightness, and posture in overhead athletes: a randomized controlled trial. Journal of sport rehabilitation, 28(3), pp.256-265. | **Population:** Athletes without scapular dyskinesis. |
| Hwang, M., Lee, S. and Lim, C., 2021. Effects of the proprioceptive neuromuscular facilitation technique on scapula function in office workers with scapula dyskinesis. Medicina, 57(4), p.332. | **Population:** Non-athlete participants. |
| Fedoriw, W.W., Ramkumar, P., McCulloch, P.C. and Lintner, D.M., 2014. Return to play after treatment of superior labral tears in professional baseball players. The American journal of sports medicine, 42(5), pp.1155-1160. | **Population:** Athletes with injury. |
| Piatti, M., Mosca, A., Omeljaniuk, R.J., Turati, M., Gaddi, D. and Bigoni, M., 2023. Comparison of isometric strength in rotator cuff and scapulothoracic muscles between elite volleyball athletes versus non-athletes. The Journal of Sports Medicine and Physical Fitness. | **Population:** Non-athlete participants. |
| Khademolhosseini N, Shadmehr A, Ghorbanpour A, Bagheri H, Jalaei S. The immediate effectiveness of whole-body vibration with the modified push-up position on neurocognitive parameters in overheads athletes with and without scapular dyskinesis. Sport sciences for health. 2023;19(3):949-57. | **Intervention:** Concurrent interventions. |
| Huang TS, Du WY, Wang TG, Tsai YS, Yang JL, Huang CY, Lin JJ. Progressive conscious control of scapular orientation with video feedback has improvement in muscle balance ratio in patients with scapular dyskinesis: a randomized controlled trial. Journal of Shoulder and Elbow Surgery. 2018;27(8):1407-14. | **Intervention:** Concurrent interventions. |
| Soliaman, R.R., Azzolini, F.L., Leme, L., Ejnisman, B., Pochini, A.D.C. and Cunha, R.A.D., 2015. The influence of training in scapular dyskinesia of volleyball players: a prospective study. Revista Brasileira de Medicina do Esporte, 21, pp.206-209. | **Outcome:** No specific pain or performance measure. |
| Cobanoglu, G., Aka, H. and Guzel, N.A., 2021. The effect of shoulder injury prevention program on glenohumeral range of motion and upper extremity performance in adolescent throwers: A pilot study. Baltic Journal of Health and Physical Activity, 13(4), p.2. | **Study type:** Pilot study. |
| Álvarez Arangua, S., Arias Poblete, LE, Espoz Lazo, S., Silva Mella, A., Paradas Osses, BY, Zamora Pinoleo, J., Rubilar Henríquez, C., Lizana Rodríguez, C., Ríos Riquelme, MA and Farías Valenzuela, C.Í., 2023. Effect of therapeutic exercise on joint range of motion and strength in throwing athletes with dyskinesia. A systematic review. | **Study type:** Review study. |
