# Supplementary material for: Effects of scapular-focused movement-based exercises on sports performance of athletes with scapular dyskinesis: A systematic review
Source: PLoS One. 2026 Apr 10;21(4):e0344540. doi: 10.1371/journal.pone.0344540 (PMC13068284; doi:10.1371/journal.pone.0344540)
Supplement: Attachment — Submitted filename: Response to editor and reviewers-Jan 2026.pdf [file pone.0344540.s004.pdf]

# Author Responses to Editors' and Reviewers' Comments

| Editor and Reviewer Comments | How and where it was addressed |
|---|---|
| **Editor** | |
| Thank you for submitting your manuscript to PLOS ONE. After careful consideration, we feel that it has merit but does not fully meet PLOS ONE's publication criteria as it currently stands. Therefore, we invite you to submit a revised version of the manuscript that addresses the points raised during the review process. | Thank you for considering our manuscript. We sincerely appreciate the constructive comments provided by the reviewers. We are pleased to confirm that we have carefully addressed all the comments and suggestions raised during the review process. The revisions have been incorporated into the manuscript, and we believe they have strengthened the paper. Thank you again for the opportunity to revise and resubmit our work. |
| **Reviewer 1** | |
| **1.** Title (Page 1, Lines 1–30)<br>**1.1** Line 1: Title lacks precision. "Movement-based exercises" is undefined and encompasses diverse interventions (stabilization, kinetic chain, suspension). Revise to "Effects of Scapular-Focused Movement-Based Exercises on Sports Performance..." | Title : Thank you for your comment. We agree that the term "movement-based exercises" was overly broad. Following your suggestion, we revised the title to clearly specify the scapular-focused nature of the intervention. The updated title now more accurately reflects the content and scope of the study.<br>Revised Title:<br>"Effects of scapular-focused movement-based exercises on sports performance of athletes with scapular dyskinesis: A systematic review" |
| **1.2** Line 14: Abstract claims "moderate-certainty evidence" for ROM improvement, but GRADE table (Page 16) rates glenohumeral ROM as moderate and scapular kinematics as low. Inconsistent summarization. | We thank the reviewer for highlighting this inconsistency in summarization. We have revised the abstract to clearly separate the findings for glenohumeral range of motion (moderate-certainty evidence) from those for scapular kinematics (low-certainty evidence). The text now accurately reflects the individual certainty ratings presented in the GRADE evidence table.<br>Revised Sentence in the Abstract:<br>"However, the evidence for pain reduction and improvement in rotator cuff/scapular strength is of low certainty, showing mixed effects depending on the specific program. Evidence for improvement in scapular kinematics is also of low certainty. " |
| **1.3** Line 25: Conclusion ("recommended for athletes") contradicts very low certainty for sports performance. Reference: Guyatt et al. (2008) – GRADE: Recommendations require high/moderate certainty. | We thank the reviewer for this valuable comment. We acknowledge that recommending interventions for athletes may be inconsistent with the very low certainty of evidence regarding sports performance. In accordance with GRADE guidance (Guyatt et al., 2008), we have revised the conclusion to accurately reflect the level of certainty:<br>Revised Conclusion:<br>"Movement-based exercises may be considered by athletes with scapular dyskinesis to potentially improve shoulder function |

| | |
|---|---|
| | and glenohumeral range of motion; however, due to very low certainty of evidence regarding pain relief, strength, and sports performance, strong recommendations cannot be made at this stage. Further well-designed, tailored RCTs are needed to clarify these effects." |
| **2.** Introduction (Pages 4–6)<br>Page 5, Line 10: Prevalence claim "61–67%" cites [3,4]; [4] is an editorial, [3] is a 2016 review. Newer data exist. Reference: Longo et al. (2022) – 70% prevalence in elite volleyball. | We thank the reviewer for prompting greater precision. We have revised the sentence on the prevalence of scapular dyskinesis to reflect newer studies (2023) that provide more nuanced, population-specific data. The updated text now states that prevalence varies widely, from 42% in asymptomatic athletes to 81% in symptomatic athletes, accurately representing the results of our analysis. |
| **2.1** Page 6, Line 1: "Comprehensive synthesis is lacking" – but similar reviews exist (e.g., Moghadam 2020 [13], Khodaverdizadeh 2023 [27]). Gap overstated. Explicitly differentiate focus on sports performance metrics, not just pain/ROM. | Thank you for your detailed comment. We have revised the sentence and gap in the manuscript:<br><br>"Despite the growing body of literature, a comprehensive synthesis focusing specifically on the impact of movement-based exercises on sports performance metrics in athletes with scapular dyskinesis is limited." |
| **2.2** The introduction is overly descriptive and, while addressing epidemiology and clinical relevance of scapular dyskinesis, fails to provide a clear theoretical pathway linking the condition to impaired sports performance. A precise biomechanical pathway (e.g., kinetic chain disruption → impaired force transfer → reduced sports performance) must be outlined, supported by classic biomechanical studies (Kibler 2013; Ludewig & Reynolds 2009). | Thank you for this constructive comment. We have revised the introduction to outline the requested biomechanical pathway, integrating concepts from Kibler (2013) and Ludewig & Reynolds (2009):<br><br>"This dysfunction is associated with compromised shoulder mechanics, which may result in pain, decreased range of motion (ROM), and potentially impaired athletic performance [6, 7]. The scapula is a critical link in the upper extremity kinetic chain, providing a stable base for glenohumeral rotation and facilitating optimal force transfer from the core and lower body to the throwing or striking arm [8-10]. Classic biomechanical models posit that scapular dyskinesis impairs this role, leading to a disruption in the kinetic chain [2, 9, 10]. Specifically, altered scapular position and motion, such as excessive anterior tilt or internal rotation, compromise glenohumeral stability and subacromial space, which is frequently associated with shoulder pain and pathology like impingement and rotator cuff tendinopathy [6, 7, 11-13]. Furthermore, this dysfunction is theorized to directly hinder sports performance by impairing the efficient generation and transfer of forces, potentially resulting in decreased throwing velocity, reduced shot power, and diminished accuracy [9, 10]. Athletes with scapular dyskinesis often report symptoms such as shoulder pain, weakness, and functional limitations, which may be associated with reduced training and competitive performance [3, 15]. " |
| **2.3** Statements such as dyskinesis may contribute to secondary shoulder pathologies imply causality. Current evidence demonstrates only association, not causation. Language must | We thank the reviewer for this important comment. We have carefully revised all relevant statements throughout the manuscript to avoid implying causality. Specifically, phrases such as dyskinesis may contribute to secondary shoulder |

| | |
|---|---|
| be tempered using terms like "is associated with" or "may play a role in the development of.<br>Hickey D, Solvig V, Cavalheri V, et al. Scapular dyskinesis increases the risk of future shoulder pain by 43% in asymptomatic athletes: a systematic review and meta-analysis. British Journal of Sports Medicine. 2018;52(2):102- 110. doi:10.1136/bjsports-2017-097559 → Only prospective association (RR = 1.43), not causation. | pathologies have been replaced with more neutral wording, including "is associated with secondary shoulder pathologies or "may play a role in the development of secondary shoulder pathologies. These changes ensure that the language accurately reflects the evidence, which demonstrates association rather than causation. |
| **2.4** Ratcliffe E, Pickering S, McLean S, et al. Is there a relationship between subacromial impingement syndrome and scapular orientation? A systematic review. Orthopaedics & Traumatology: Surgery & Research. 2014;100(6):619-626. doi:10.1016/j.otsr.2014.05.008 → Concludes: "Correlation does not imply causation"; confounding by training volume, age, etc. | We thank the reviewer for highlighting this reference. Following Ratcliffe et al. (2014), we have clarified throughout the manuscript that the observed relationships between scapular dyskinesis and subacromial impingement syndrome reflect associations only, and do not imply causation. Potential confounding factors such as training volume, age, and other variables are now acknowledged in the relevant sections |
| **2.5** The introduction claims sports performance outcomes (throwing velocity, agility, upper-limb stability) will be evaluated, but provides no preliminary evidence linking dyskinesis correction to actual performance gains. This gap must be acknowledged, given the scarcity of studies. | Thank you for mentioning this. We have updated the introduction and have added these sentences to it:<br><br>"However, it is important to acknowledge that direct, high-level evidence linking the specific correction of scapular dyskinesis to measurable gains in these sports performance outcomes remains scarce, representing a significant gap in the current literature." |
| **2.6** The claim "a comprehensive synthesis… on sports performance is lacking" is overstated. Prior reviews (Cools 2014; Hickey 2018) have partially addressed this. The specific gap and added value of this review must be clearly defined.<br><br>Cools AM, Struyf F, De Mey K, et al. Rehabilitation of scapular dyskinesis: from the office worker to the elite overhead athlete. British Journal of Sports Medicine. 2014;48(8):692-697. doi:10.1136/bjsports-2013-092299 → Focus: clinical outcomes; performance mentioned only anecdotally.<br><br>Hickey D, Solvig V, Cavalheri V, et al. Scapular dyskinesis increases the risk of future shoulder pain…British Journal of Sports Medicine. 2018;52(2):102-110. doi:10.1136/bjsports-2017-097559 → Risk-focused; no intervention synthesis on performance. | Thank you. We have updated some sections based on your comment:<br><br>"Despite the growing body of literature, the existing syntheses addressing the impact of movement-based exercises on sports performance in athletes with scapular dyskinesis remain limited in scope and consistency. Although previous reviews have partially explored performance-related or clinical aspects [14, 29], their primary emphasis has largely been on clinical and biomechanical outcomes rather than on systematic evaluation of sport-specific performance measures.<br>Most prior reviews have emphasized clinical outcomes such as pain and disability, but few have systematically examined whether these improvements translate into measurable changes in sports performance parameters, such as throwing velocity, agility, and upper-limb stability [22, 30]. Previous reviews have often focused on specific exercise protocols or individual outcomes, leaving uncertainty regarding the broader and comparative implications of different movement-based interventions on performance-related variables [15, 26]. " |
| **2.7** Inconsistent terminology: "dyskinesia" and "dyskinesis" are used interchangeably. The standard scientific term is scapular dyskinesis. Please standardize throughout. 3. Methods (Pages 6–11) | We thank the reviewer for this comment. The terminology has been standardized throughout the manuscript, and "scapular dyskinesis" is now used consistently. |

| | |
|---|---|
| **3.** Methods (Pages 6–11)<br>**3.1** Search Strategy (Page 7, Table 1): Search limited to 3 databases. Embase, CINAHL, SportDiscus missing. Reference: Cochrane Handbook comprehensive search requires ≥4 databases. | We thank the reviewer for this comment. Due to limited access, Embase, CINAHL, and SportDiscus could not be included. However, the search strategy still covers major databases to ensure comprehensive literature retrieval. Also, reference lists of included articles were screened to identify additional relevant studies, supplemented by a grey literature search via Google Scholar. The Connected Papers platform was also utilized to capture any other pertinent publications (https://www.connectedpapers.com/). |
| **3.2** Eligibility Criteria (Page 8, Table 2) Intervention criterion: movement-based" – but includes suspension training, mirror cross, PSSE. Operational definition absent. | We thank the reviewer for highlighting this issue. We acknowledge that the term "movement-based intervention" was not clearly defined in the eligibility criteria. To address this, we have now added an explicit operational definition in Section 3.2 and clarified Table 2.<br>Movement-based interventions: Interventions in which the therapeutic effect is primarily achieved through active, voluntary movement performed by the participant, involving muscle activation, postural control, or neuromuscular retraining, rather than passive techniques." Based on this definition, suspension training, mirror-cross training, and PSSE were appropriately included, as all require active execution of movement patterns and neuromuscular engagement. |
| **3.3** Comparator: "no exercise or placebo" – yet studies with "usual training" or "multimodal PT" included. Criterion violation. Example: Wen et al. (2025) compares SDBET vs. MPT → both active. | Thank you for this important observation. We acknowledge the need for greater clarity in our original description of the comparator. Our intention was to include studies in which the primary experimental comparison was between a scapular-focused movement-based exercise protocol and an alternative condition that did not specifically target scapular dyskinesis within the defined movement-based paradigm. This includes both passive controls (e.g., no exercise, placebo modalities such as sham ultrasound) and active control conditions, such as "usual training," "standard physical therapy," or "multimodal PT," that represent the standard of care or a non-specialized alternative. In the example you cite (Wen et al., 2025), the "Multimodal Physical Therapy (MPT)" group serves as the control/comparator condition against the specialized "Scapular Dynamic-Balanced Exercise Training (SDBET)." This aligns with our PICO framework, as the MPT group functions as the comparative intervention against which the specific experimental protocol is evaluated. We agree that our original phrasing ("no exercise or a placebo intervention") was overly restrictive and did not accurately convey this scope.<br>For methodological precision, we have revised the manuscript to define the comparator more clearly as "a control condition (e.g., no intervention, placebo, usual care, or an alternative non-scapular-focused rehabilitation protocol." This clarifies that studies with an active comparison group were included if that group did not involve the specific movement-based intervention under investigation. We appreciate your feedback, which |

| | |
|---|---|
| | allows us to correct this terminology and present our selection criteria more transparently. |
| **3.4** Page 15, Table 5: Sports performance: only 2 RCTs (n=64) → very low certainty justified, but recommendation in conclusion (Page 23) contradicts. | We sincerely thank the reviewer for this valuable comment. The necessary revisions have been made, and the conclusion has been updated to align with the level of evidence. |
| **3.5** Publication bias not assessed (funnel plot infeasible) – must be stated as limitation. | Thank you. We have included this in the limitations.<br><br>"Publication bias could not be formally assessed because the number of included studies per outcome was insufficient to generate funnel plots, which may have affected the certainty of the findings." |
| **3.6** The search strings presented in Table 1 are comprehensive but lack sufficient sensitivity. Key terms such as "stabilization" and "kinetic chain" are missing, which may have led to omission of relevant studies. | Thank you for this constructive feedback regarding the search strategy's sensitivity. We appreciate the opportunity to clarify our methodological choices. The development of our search strings followed an iterative process aimed at balancing sensitivity with precision. While the terms suggested are indeed relevant to the field, our pilot searches indicated that incorporating broader conceptual terms such as "stabilization" or "kinetic chain" without strict contextual linkage to the scapula significantly increased the volume of irrelevant records, reducing the overall efficiency and specificity of the search. Our final strategy was therefore deliberately structured to capture studies through a combination of focused, high-yield terms directly related to our PICO components:<br><br>The population (athlet*, sport*),<br>The intervention (exercis*, rehabilitat*),<br>The outcomes (perform*, strength*, function*),<br>The condition ("scapular dyskinesis", scapul*).<br><br>We are confident that this targeted approach, combined with our supplementary search methods (e.g., hand-searching reference lists of included studies and relevant reviews), has effectively captured the body of literature pertinent to our specific research question. To address your concern and enhance methodological transparency, we can revise the manuscript to include a brief justification for selecting key terms, acknowledging that broader, related concepts were omitted to maintain search precision. |
| **3.7** The grey literature search (Google Scholar and Connected Papers) is insufficient. No manual search of conference proceedings, ClinicalTrials.gov, or expert consultation was conducted. | Thank you for the comment. We acknowledge that a more extensive grey literature search, including specific registries, could be beneficial. Our supplementary search strategy, comprising reference list screening and the use of Connected Papers/Google Scholar, was designed to mitigate the risk of omitting key studies within the scope of this review. We have |

| | |
|---|---|
| | revised the manuscript text to more precisely describe the methods employed. |
| **3.8** PICOS violations: Population: Studies including participants with concurrent injuries (e.g., SIS in Wen 2025) were included. Comparator: The eligibility criterion states "no exercise/placebo", yet studies with active controls (e.g., massage in Nowotny 2018) were included. Outcome: Some included studies did not report primary outcomes such as VAS or NRS. Apply the PICOS criteria strictly and provide justification for any included studies that violate these criteria. | Thank you for your comment regarding the comparator. A detailed clarification has been provided in our response to comment 3.3.<br>Thank you for this important observation about the Outcome. We applied the PICOS criteria strictly during the study selection process. Our defined outcome domain for pain specifically required assessment via VAS or NRS. Studies that did not assess pain using these specific tools were not included based on the pain outcome. However, studies were retained if they fully met all other PICOS criteria and reported on our other primary outcome: objective sport-performance metrics (e.g., velocity, jump height, muscle activity). In these cases, the study contributed to the synthesis of performance outcomes but not to the analysis of pain. Revised section in the PICOS table:<br><br>"Studies were required to report on at least one primary outcome: (1) pain intensity assessed specifically via the Visual Analog Scale or Numeric Rating Scale, or (2) objective sport-performance metrics (e.g., throwing velocity, jump height, electromyographic measures)." |
| **4.** Results (Pages 11–19)<br>**4.1** Table 3 (Pages 12–14): SD type = NA in 8/14 studies. How was dyskinesis diagnosed? Examples: Wen (2025), Karimi (2024) – no diagnostic tool (SDT? Visual observation?). | We thank the reviewer for this important observation. In several included studies, scapular dyskinesis was confirmed but not classified into specific subtypes, resulting in "NR" entries in Table 3. Where reported, diagnosis was based on visual observation or clinical assessment tools. Some studies did not specify the exact diagnostic method. We have now acknowledged this lack of standardized diagnostic criteria as a source of heterogeneity in the Results and Discussion sections. |
| **4.2** Exercise intensity NA in most studies. How was SWiM synthesis conducted? Conducting a SWiM synthesis without dose–response data is not feasible; therefore, the clinical reproducibility is effectively very low. | We thank the reviewer for this insightful comment. We acknowledge that exercise intensity was not reported in most included studies, which limits the ability to conduct a traditional dose–response synthesis. The SWiM approach was applied to qualitatively summarize intervention effects across studies, focusing on the presence or absence of outcomes rather than precise exercise dose. We have now explicitly stated in the Methods and Discussion sections that the lack of intensity reporting limits clinical reproducibility and generalizability |
| **4.3** Merolla et al. 2010 a/b/c: Three entries from same sample? Duplicate reporting. Should be merged into one study with multiple outcomes. | We thank the reviewer for this comment. While Merolla et al. (2010 a/b/c) report outcomes from the same overall study, each entry represents a slightly different subgroup with minor differences in sample size, sex distribution, and mean age. To maintain accuracy and transparency, we have kept all three entries in Table 3 and added a footnote clarifying these differences. |

| | |
|---|---|
| **4.4** Page 17, Line 10: "Consistent improvements in IR ROM" – but Ilyoung (2018) reports decreased ER ROM and increased GIRD in SSE group. Contradictory. | Thank you for your constructive comment. Explanation of Changes:<br>• Narrative text:<br>Changed: "Improvement in ROM was a consistent finding..." to "Improvement in ROM, particularly for internal rotation, was a common finding..." This more precise language avoids overgeneralization. Added: "...though the effects on external rotation and the overall pattern of adaptation varied." This explicitly acknowledges the variability highlighted by the reviewer. Revised the description of Ilyoung et al. to clarify the complex finding: It now explicitly states the concurrent increase in IR and decrease in ER, correctly frames the net decrease in GIRD as an outcome, and interprets this as a "targeted shift in rotational balance." This accurately captures the study's results without contradiction.<br><br>• GRADE Table:<br>Changed: "Consistent improvements in IR (and ER in some)..." to "Generally positive effects on IR ROM... Effects on ER ROM were less consistent, with one study showing a decrease..." |
| **4.5** Page 18, Table 4: "No significant effect" column empty for 7 outcomes. Why? (Only 4 studies for function). Misleading. | We thank the reviewer for this important comment. The "No significant effect" column in Table 4 was empty for 7 outcomes because none of the included studies reported non-significant findings for these outcomes. Only 4 studies assessed shoulder function, but for the remaining outcomes, all reported significant effects or did not report data suitable for this column. We have now added a clarifying footnote to Table 4 to prevent misinterpretation |
| **4.6** Table 5 and results: Sport performance outcomes are based on only two RCTs (n = 64), yielding very low certainty of evidence (Table 5). However, the manuscript concludes that the intervention 'is recommended for athletes,' which contradicts GRADE guidance. | We thank the reviewer for highlighting this point. The manuscript has been revised to reflect that sport performance outcomes are based on only two RCTs (n = 64), yielding very low certainty of evidence. The conclusion now clarifies that while movement-based interventions may have potential benefits, the current evidence is insufficient to make firm recommendations for athletes |
| **5.** Discussion & Conclusion (Pages 19–23)<br>**5.1** Page 22, Line 5: "Structured scapular-focused exercise can be considered" – irresponsible with very low certainty for performance. Reference: GRADE Handbook – recommendations prohibited with very low certainty. | We thank the reviewer for this important comment. In accordance with the GRADE Handbook, we have revised the Discussion and Conclusion to avoid suggesting recommendations for sport performance outcomes with very low certainty. The text now emphasizes that evidence is currently insufficient to support firm recommendations, while highlighting potential benefits of structured scapular-focused exercises for other outcomes (e.g., ROM, strength, pain) |
| **5.2** There is no subgroup or sensitivity analysis exploring heterogeneity. The authors could have examined, for example, RCT-only studies, overhead-sport athletes only, or type-I | We thank the reviewer for this insightful comment. We acknowledge that the small number of studies and heterogeneity in designs, populations, and interventions limited |

| | |
|---|---|
| interventions only. At present, no investigation of heterogeneity has been conducted, which limits the interpretability of the findings. | our ability to conduct subgroup or sensitivity analyses, which affects the interpretability and generalizability of the findings |
| **5.3** Future directions suggest "standardized protocols" but authors provide none. | Thank you for the feedback. The authors acknowledge that while future directions emphasize standardized protocols to enhance reproducibility and comparability across studies, the current work focuses on preliminary findings from an exploratory phase rather than prescriptive guidelines. Detailed protocols will be developed and validated in subsequent prospective trials, building on the methodological framework outlined in the methods section, with plans to disseminate them via open-access repositories for broader adoption. This approach aligns with best practices in rehabilitation research, where standardization evolves iteratively from initial evidence |
| **Reviewer 2** | |
| **1.** In terms of clarity, in the introduction the rationale for specifically linking movement-based exercise to sport-specific performance is not sufficiently developed. Much of the introduction focuses on clinical outcomes (pain, ROM, disability), whereas the objective emphasizes sports performance metrics. The conceptual pathway from dyskinesis → biomechanical impairment → decreased performance should be more explicitly synthesized from current literature. Add a paragraph that explicitly connects scapular mechanics to performance metrics such as throwing velocity, power, stability, and proprioception. | Thank you for this comment. We have revised the introduction to explicitly detail the conceptual pathway from scapular dyskinesis to biomechanical impairment and its potential impact on key sports performance metrics like velocity and power.<br><br>"The scapula is a critical link in the upper extremity kinetic chain, providing a stable base for glenohumeral rotation and facilitating optimal force transfer from the core and lower body to the throwing or striking arm [8-10]. Classic biomechanical models posit that scapular dyskinesis impairs this role, leading to a disruption in the kinetic chain [2, 9, 10]. Specifically, altered scapular position and motion, such as excessive anterior tilt or internal rotation, compromise glenohumeral stability and subacromial space, which is frequently associated with shoulder pain and pathology like impingement and rotator cuff tendinopathy [6, 7, 11-13]. Furthermore, this dysfunction is theorized to directly hinder sports performance by impairing the efficient generation and transfer of forces, potentially resulting in decreased throwing velocity, reduced shot power, and diminished accuracy [9, 10]. " |
| **2.** The search strategy is extensive, but the PICOS criteria reveal conceptual issues. Outcome criteria requiring both pain and performance measures may have excluded relevant RCTs. Inclusion of acute and long-term interventions introduces major heterogeneity. Clarify whether studies needed both pain and performance outcomes and justify inclusion of single-session studies. | We sincerely thank the reviewer for this insightful comment, which helped us clarify the inclusion criteria regarding pain and performance outcomes, as well as the rationale for including single-session studies. Also, the PICOS table has been updated. |
| **3.** Although RoB-2 and ROBINS-I were applied, several inconsistencies are present. Some studies lacking allocation | We sincerely thank the reviewer for this precise, insightful, and constructive comment. We fully agree that exercise-based trials |

| | |
|---|---|
| concealment or blinding are rated as low measurement bias despite subjective outcomes. Provide more detailed justification for each bias domain and acknowledge the high likelihood of performance/detection bias in exercise trials. | are inherently susceptible to performance and detection bias, particularly because blinding participants and therapists is often impractical. The reviewer's careful attention to this important methodological challenge is highly appreciated.<br>Regarding the application of the RoB-2 tool, it should be noted that this instrument is a domain-based, probability-oriented algorithm, in which final judgments are derived from the overall body of available methodological evidence rather than from the presence or absence of any single methodological feature.<br>Specifically, within the randomization process, studies that did not explicitly report allocation concealment were classified as having a low risk of bias when baseline characteristics between intervention groups showed no statistically or clinically meaningful differences. This approach is consistent with the RoB-2 framework's guidance.<br>In the domain of outcome measurement, although certain outcomes (e.g., pain intensity) were subjective and self-reported, these variables were assessed using standardized, validated instruments, and outcome assessors were not involved in data recording. Consequently, the absence of assessor blinding in these cases was considered unlikely to introduce systematic measurement bias.<br>Furthermore, even in studies where formal blinding was not implemented, the presence of methodological safeguards, such as randomized allocation, standardized intervention protocols, and the absence of baseline group differences, was judged to reduce the likelihood of bias arising from deviations from intended interventions or from outcome assessment. Accordingly, these domains were rated as low risk of bias when supported by the overall methodological context.<br>Finally, as emphasized in the RoB-2 guidance, risk-of-bias judgments may also take into account contextual and indirect indicators, including consistency of reporting, methodological transparency, study conduct, and, when necessary, an informed probabilistic judgment based on the comprehensive evaluation of each article. In this regard, all available information from each included study was carefully considered during the risk-of-bias assessment process. |
| **4.** The GRADE table contains inconsistencies. Moderate certainty ratings for ROM and function are questionable given small samples and heterogeneity. Reassess GRADE downgrades for risk of bias and inconsistency. | Thank you for this methodological point. We have reassessed the GRADE downgrades for the outcomes of function and ROM. While sample sizes remain small (justifying the imprecision downgrade), we maintain that a further downgrade for inconsistency is not warranted, as the variability in results is explainable and expected due to differences in intervention protocols and specific outcome measures. We have revised the |

| | |
|---|---|
| | GRADE table to provide a more detailed justification for each domain assessment, clarifying why the certainty was rated as moderate. |
| **5.** The decision not to provide a meta-analysis should be justified better. Several outcomes (e.g., IR ROM, ER strength, VAS) are conceptually identical and potentially meta-analyzable.Eitherprovide clear methodological justification or conduct small meta-analyses | We sincerely thank the reviewer for this insightful comment regarding the potential for meta-analysis, which helped us clarify our methodological decisions. Accordingly, the text has been revised to explicitly explain why a meta-analysis was not performed and why a narrative synthesis (SWiM) was applied, including details on heterogeneity in outcomes, measurement protocols, timing, and participant characteristics |
| **6.** Only two studies include true sport-specific performance metrics. The Discussion extrapolates performance improvements from clinical improvements, which is speculative. I think that these should be excluded from your study since they are completely different populations and aims. | Thank you for this important comment. We have revised the Discussion to directly address your concern. We now explicitly acknowledge that only two included studies measured sport-specific performance metrics, and we have added clear statements throughout to caution against extrapolating clinical improvements to confirmed performance gains. The limitations and future directions sections have been strengthened to highlight this critical evidence gap. |
| **7.** Sections 4.1–4.3 contain repeated explanations and mechanisms. Condense the Discussion to focus on key certainties, implications and research needs. | Thank you for the suggestion. We have condensed Sections 4.1–4.3. |
| **8.** Some in-text citations require correction to meet PLOS ONE requirements. Check again the references. | The entire manuscript regarding this has been updated. |
| **9.** Address minor stylistic and grammatical issues to improve clarity. | Thank you for your comment. The whole draft has been checked right now. |
| **10.** The PROSPERO registration number (CRD42025635493) appears in the manuscript; however, upon inspection, the registered title “How long should athletes stretch? acute stretching durations for flexibility and performance: a systematic review and meta-analysis” is entirely unrelated to the present manuscript, which focuses on scapular dyskinesis and movement-based rehabilitation-specified methodology, and potential selective reporting. PROSPERO requires that systematic reviews follow the protocol outlined at registration or clearly justify any deviations. In this case, the topic, population, outcomes, and intervention categories differ completely from the registered protocol, suggesting that either the wrong registration number was inserted or the review did not follow a prospectively registered plan. | We sincerely thank the reviewer for highlighting this issue. The PROSPERO registration number has now been corrected to CRD420251135347. |
