# Supplementary material for: Effects of scapular-focused movement-based exercises on sports performance of athletes with scapular dyskinesis: A systematic review
Source: PLoS One. 2026 Apr 10;21(4):e0344540. doi: 10.1371/journal.pone.0344540 (PMC13068284; doi:10.1371/journal.pone.0344540)
Supplement: Attachment — Submitted filename: Dr,moh.docx [file pone.0344540.s003.docx]

Dear Dr. Mohsenifar,

I have carefully reviewed the manuscript titled “**The effects of movement-based exercises on sports performance of athletes with scapular dyskinesis: A systematic review**” and provide the following detailed comments for your consideration.

The manuscript addresses an important topic, but significant methodological, reporting, and interpretive issues limit the reliability and applicability of its conclusions. Major revisions are required before it can be considered for publication.

Sincerely,

1. Title & Abstract (Page 1, Lines 1–30)

Line 1: Title lacks precision. "Movement-based exercises" is undefined and encompasses diverse interventions (stabilization, kinetic chain, suspension). Revise to *"Effects of Scapular-Focused Movement-Based Exercises on Sports Performance..."*

- Line 14: Abstract claims "moderate-certainty evidence" for ROM improvement, but GRADE table (Page 16) rates glenohumeral ROM as moderate and scapular kinematics as low. Inconsistent summarization.
- Line 25: Conclusion ("recommended for athletes") contradicts *very low* certainty for sports performance. Reference: Guyatt et al. (2008) – GRADE: Recommendations require high/moderate certainty.

2. Introduction (Pages 4–6)

- Page 5, Line 10: Prevalence claim "61–67%" cites [3,4]; [4] is an editorial, [3] is a 2016 review. Newer data exist. Reference: Longo et al. (2022) – 70% prevalence in elite volleyball.
- Page 6, Line 1: "Comprehensive synthesis is lacking" – but similar reviews exist (e.g., Moghadam 2020 [13], Khodaverdizadeh 2023 [27]). Gap overstated. Explicitly differentiate focus on sports performance metrics, not just pain/ROM.
- The introduction is overly descriptive and, while addressing epidemiology and clinical relevance of scapular dyskinesis, fails to provide a clear theoretical pathway linking the condition to impaired sports performance. A precise biomechanical pathway (e.g., kinetic chain disruption → impaired force transfer → reduced sports performance) must be outlined, supported by classic biomechanical studies (Kibler 2013; Ludewig & Reynolds 2009).
- 2. Statements such as “dyskinesis may contribute to secondary shoulder pathologies” imply causality. Current evidence demonstrates only association, not causation. Language must be tempered using terms like “is associated with” or “may play a role in the development of.”
- Hickey D, Solvig V, Cavalheri V, et al. Scapular dyskinesis increases the risk of future shoulder pain by 43% in asymptomatic athletes: a systematic review and meta-analysis. *British Journal of Sports Medicine*. 2018;52(2):102-110. doi:10.1136/bjsports-2017-097559 → Only prospective association (RR = 1.43), not causation.
- Ratcliffe E, Pickering S, McLean S, et al. Is there a relationship between subacromial impingement syndrome and scapular orientation? A systematic review. *Orthopaedics & Traumatology: Surgery & Research*. 2014;100(6):619-626. doi:10.1016/j.otsr.2014.05.008 → Concludes: "Correlation does not imply causation"; confounding by training volume, age, etc.
- The introduction claims sports performance outcomes (throwing velocity, agility, upper-limb stability) will be evaluated, but provides no preliminary evidence linking dyskinesis correction to actual performance gains. This gap must be acknowledged, given the scarcity of studies.
- The claim “a comprehensive synthesis… on sports performance is lacking” is overstated. Prior reviews (Cools 2014; Hickey 2018) have partially addressed this. The specific gap and added value of this review must be clearly defined.
- Cools AM, Struyf F, De Mey K, et al. Rehabilitation of scapular dyskinesis: from the office worker to the elite overhead athlete. *British Journal of Sports Medicine*. 2014;48(8):692-697. doi:10.1136/bjsports-2013-092299 → Focus: clinical outcomes; performance mentioned only anecdotally.
- Hickey D, Solvig V, Cavalheri V, et al. Scapular dyskinesis increases the risk of future shoulder pain…*British Journal of Sports Medicine*. 2018;52(2):102-110. doi:10.1136/bjsports-2017-097559 → Risk-focused; no intervention synthesis on performance.
- Inconsistent terminology: “dyskinesia” and “dyskinesis” are used interchangeably. The standard scientific term is scapular dyskinesis. Please standardize throughout.

3. Methods (Pages 6–11)

3.1 Search Strategy (Page 7, Table 1): Search limited to 3 databases. Embase, CINAHL, SportDiscus missing. Reference: Cochrane Handbook – comprehensive search requires ≥4 databases.

3.2 Eligibility Criteria (Page 8, Table 2)

- Intervention criterion: "movement-based" – but includes suspension training, mirror cross, PSSE. Operational definition absent.
- Comparator: "no exercise or placebo" – yet studies with "usual training" or "multimodal PT" included. Criterion violation.

Example: Wen et al. (2025) compares SDBET vs. MPT → both active.

Data Extraction (Page 9): Authors contacted for missing data" – outcome of contact not reported. How many studies had missing data?

- Page 15, Table 5: Sports performance: only 2 RCTs (n=64) → very low certainty justified, but recommendation in conclusion (Page 23) contradicts.
- Publication bias not assessed (funnel plot infeasible) – must be stated as limitation.

The search strings presented in Table 1 are comprehensive but lack sufficient sensitivity. Key terms such as *“stabilization”* and *“kinetic chain”* are missing, which may have led to omission of relevant studies.

The grey literature search (Google Scholar and Connected Papers) is insufficient. No manual search of conference proceedings, ClinicalTrials.gov, or expert consultation was conducted.

PICOS violations: Population: Studies including participants with concurrent injuries (e.g., SIS in Wen 2025) were included. Comparator: The eligibility criterion states *“no exercise/placebo”*, yet studies with active controls (e.g., massage in Nowotny 2018) were included. Outcome: Some included studies did not report primary outcomes such as VAS or NRS. Apply the PICOS criteria strictly and provide justification for any included studies that violate these criteria.

Results (Pages 11–19)

Table 3 (Pages 12–14): SD type = NA in 8/14 studies. How was dyskinesis diagnosed?

Examples: Wen (2025), Karimi (2024) – no diagnostic tool (SDT? Visual observation?).

- Exercise intensity NA in most studies. How was SWiM synthesis conducted? Conducting a SWiM synthesis without dose–response data is not feasible; therefore, the clinical reproducibility is effectively very low.
- Merolla et al. 2010 a/b/c: Three entries from same sample? Duplicate reporting. Should be merged into one study with multiple outcomes.
- Page 17, Line 10: "Consistent improvements in IR ROM" – but Ilyoung (2018) reports decreased ER ROM and increased GIRD in SSE group. Contradictory.
- Page 18, Table 4: "No significant effect" column empty for 7 outcomes. Why? (Only 4 studies for function). Misleading.
- Table 5 and results: Sport performance outcomes are based on only two RCTs (n = 64), yielding *very low* certainty of evidence (Table 5). However, the manuscript concludes that the intervention ‘is recommended for athletes,’ which contradicts GRADE guidance

5. Discussion & Conclusion (Pages 19–23)

- Page 22, Line 5: "Structured scapular-focused exercise can be considered" – irresponsible with *very low* certainty for performance. Reference: GRADE Handbook – recommendations prohibited with very low certainty.
- There is no subgroup or sensitivity analysis exploring heterogeneity. The authors could have examined, for example, RCT-only studies, overhead-sport athletes only, or type-I interventions only. At present, no investigation of heterogeneity has been conducted, which limits the interpretability of the findings.
- Future directions suggest "standardized protocols" – but authors provide none.
